# Amelioration of Atherosclerosis by lycopene is linked to the modulation of gut microbiota dysbiosis and related gut-heart axis activation in high-fat diet-fed ApoE^−/−^ mice

**DOI:** 10.1186/s12986-023-00772-x

**Published:** 2023-12-01

**Authors:** Tengcan Tu, Hao Liu, Zhenhao Liu, Yunyi Liang, Chujun Tan, Dan Feng, Jun Zou

**Affiliations:** 1 The Second School of Clinical Medicine, Southern Medical University, Guangzhou, 510280 China; 2 Department of Cardiology, The Sixth Affiliated Hospital, School of Medicine, South China University of Technology, 120 Guidan Road, Foshan, 528200 Guangdong Province China; 3 Department of Cardiology, The Seventh Affiliated Hospital of Southern Medical University, Foshan, 528244 China; 4 Department of Cardiology, Ganzhou People’s Hospital, Ganzhou, 341000 China; 5 Health Management Center, The Sixth Affiliated Hospital, School of Medicine, South China University of Technology, Foshan, 528200 China; 6 Guangdong Provincial Key Laboratory of Food, Nutrition and Health, Department of Nutrition, School of Public Health, Sun Yat-sen University, Guangzhou, 510080 China

**Keywords:** Lycopene, Atherosclerosis, Gut microbiota, Toll-like receptor 4, Nuclear factor-κB

## Abstract

**Background:**

Interplay between gut microbiota and heart, termed “gut-heart” axis, has a crucial role in the pathogenesis of atherosclerosis. Our previous study showed that lycopene possesses anti-inflammatory and anti-atherosclerotic effects, but its link to the gut microbiota is poorly understood. Herein, we surmised that lycopene could regulate the gut microbiota, exert anti-atherosclerotic effect by regulating the “gut-heart” axis.

**Methods:**

Male ApoE^−/−^ mice were fed a high-fat diet (HFD) with or without lycopene (0.1% w/w) for 19 weeks. Gut microbiota was analyzed by 16 S rRNA sequencing, the protein levels of zonula occludens-1 (ZO-1), occludin, toll-like receptor 4 (TLR4) and phospho-nuclear factor-κB (NF-κB) p65 were measured by Western blotting, the levels of serum inflammatory factors including monocyte chemotactic protein 1 (MCP-1), tumor necrosis factor-α (TNF-α), interleukin-1β (IL-1β), and IL-6 were assayed using ELISA kits. Also, the concentrations of serum lipopolysaccharide (LPS), D-lactic acid (D-LA) and diamine peroxidase (DAO) were measured through ELISA method.

**Results:**

The aortic sinus sections revealed that lycopene supplementation significantly reduced the extent of atherosclerotic lesions and inhibited atherosclerosis development caused by HFD. The analysis of gut microbiota showed that lycopene reduced the ratio of Firmicutes/Bacteroides and increased the relative abundance of Verrucomicrobia, *Akkermansia* and *Alloprevotella*, which were related to elevated intestinal barrier function and reduced inflammation. Moreover, lycopene up-regulated the expression of intestinal ZO-1 and occludin and decreased serum LPS, D-LA and DAO levels. In addition, lycopene inhibited the expression of TLR4 and phospho-NF-κB p65 in aortic sinus plaque, serum MCP-1, TNF-α, IL-1β, and IL-6 levels were also lowered by lycopene treatment.

**Conclusions:**

Our results indicated the protective effect of lycopene against atherosclerosis induced by HFD and further revealed that its mechanism might be its prebiotic effect on maintaining gut microbiota homeostasis and improving intestinal barrier function, consequently reducing serum LPS-triggered inflammatory response in the heart.

## Introduction

Atherosclerosis and its related cardiovascular diseases still have an extremely high morbidity and mortality rate globally, despite remarkable advances in their prevention, diagnosis and treatment [1]. The pathological signs of atherosclerosis consist of lipid infiltration and plaque formation in arteries. However, the molecular mechanism of atherosclerotic plaque formation has not yet been determined, although studies have linked it to genetics, diet, environmental factors, inflammation and autoimmune disorders [2, 3].

Emerging evidence has reported a strong association between the gut microbiota dysbiosis and the development of atherosclerosis, thus the concept of the “gut-heart” axis has been proposed [4, 5]. A high-fat diet (HFD) is a well-established risk factor for atherosclerosis. Recent research has shown that alterations in gut microbial diversity caused by HFD are directly related to the pathogenesis of atherosclerosis, which is initiated through the activation of the gut-heart axis [6]. Gut microbiota dysbiosis can impair the intestinal barrier function by disrupting intestinal tight junction proteins including zonula occludens-1 (ZO-1) and occludin, thereby increasing intestinal permeability and allowing more lipopolysaccharide (LPS) to enter the circulation, which in turn stimulates the toll-like receptor 4 (TLR4)/nuclear factor-kappa B (NF-κB) signal cascades, thus activating various inflammatory factors and potentially resulting in atherosclerosis [5]. The atherosclerotic apolipoprotein E knockout (ApoE^−/−^) mice showed significantly increased intestinal permeability due to the disruption of intestinal tight junction proteins [7], suggesting that intestinal barrier function might also be crucial in the development of atherosclerosis.

Lycopene is a type of fat-soluble carotenoid found in red fruits such as tomatoes, apricots, papayas, and watermelons [8]. In Western diets, lycopene consumption is high, with a total intake of nearly 2~5 mg/d, and the physiological content of lycopene is approximately 1~2 µmol/L [8, 9]. According to multiple research, lycopene has antioxidant, anti-inflammatory and lipid-lowering properties [8–11]. Dietary lycopene supplementation has also been found to improve gut microbiota dysbiosis and intestinal barrier function in weaned piglets and alleviate stress from early weaning [12]. In addition, epidemiologic research has demonstrated a negative correlation between serum lycopene concentration and atherosclerosis [13]. Supplementation with lycopene has also been shown to protect against HFD-induced atherosclerosis in ApoE^−/−^ mice [14]. The underlying anti-atherogenic mechanism of lycopene may be related to its antioxidant, anti-inflammatory and lipid-lowering effects [9]. Since the development of atherosclerosis is also influenced by the gut microbiota dysbiosis, we, herein, addressed a question whether lycopene can achieve this anti-atherosclerotic effect by inhibiting gut microbiota dysbiosis and consequently reducing serum LPS-triggered inflammatory response in the heart and suppressing atherosclerosis.

Therefore, we hypothesized that lycopene could effectively protect gut microbiota dysbiosis induced by the high-fat diet, reduce intestinal permeability and blood LPS levels, thereby inhibiting cardiovascular TLR4/NF-κB signal cascade and preventing the progression of atherosclerosis. To that end, we selected ApoE^−/−^ mice that were given a long-term high-fat diet, which is the most commonly used animal model to simulate atherosclerosis.

## Materials and methods

### Animal model and treatment

Male ApoE^−/−^ mice at the age of six weeks were provided by Vital River Laboratory Animal Technology Co. Ltd (Beijing, China). The animals were maintained in a SPF grade and regulated environment (temp. 22 ± 2℃ and a 12-hour day-night cycle). All mice were first given a standard chow diet for a week before being randomly allocated to one of three groups: low-fat diet (Control group, n = 8), high-fat diet (HFD group, n = 8), high-fat diet supplemented with 0.1% w/w lycopene (HFD + LY group, n = 8) for 19 weeks. Table 1 lists the diets’ constituents. As previously described [15], fecal samples were collected for each mouse separately and stored at -80 °C for extracting DNA profile of the gut microbiota. After an overnight fast, mice were anaesthetized with pentobarbital and sacrificed. Orbital blood was collected to obtain serum. Blood specimen, small intestine, aortas, and the entire heart were collected, separated and stored at -80℃ until needed. After that, 1mL of lysis buffer was used to dissolve the scraped intestinal mucosa.

**Table 1:** Ingredient composition proportion (%) of the experimental diets fed to ApoE^−/−^ mice

	Group		
Ingredient	Control	HFD	HFD + LY
Casein	19.47%	19.47%	19.47%
Corn starch	21.17%	4.99%	4.99%
Maltodextrin	9.98%	9.98%	9.98%
Sucrose	34.04%	34.11%	34.01%
Cellulose	4.99%	4.99%	4.99%
Corn oil	1.28%	0.99%	0.99%
Anhydrous milk fat	3.72%	19.97%	19.97%
Cholesterol	0	0.15%	0.15%
Mineral and Vitamin premix	5.35%	5.35%	5.35%
Lycopene	0	0	0.10%
Energy per gram of feed	3.89 kcal	4.70 kcal	4.70 kcal
Fat calories	11%	41%	41%
Protein calories	20%	17%	17%
Carbohydrate calories	69%	43%	43%

The name of the diet and source, manufacturer:

Control, MD12014; HFD, MD12015; HFD + LY, MD12015 supplemented with lycopene; Medicience Ltd,China.

Control, low-fat diet; HFD, high-fat diet; HFD + LY, high-fat diet supplemented with lycopene.

The experimental animals procedures were conducted in accordance with the standards set by the Institutional Animal Care and Use Committee (IACUC) office of Sun Yat-sen University and was approved by them (No. 2019-035).

### Biochemical analysis

Blood total cholesterol (TC), triglycerides (TG), low density lipoprotein cholesterol (LDL-C) and high density lipoprotein cholesterol (HDL-C) were assessed using the relevant enzymatic kits (Jiancheng, Nanjing, China) according to the kits instructions.

### Enzyme-linked immunosorbent assay (ELISA)

Serum LPS, D-lactic acid (D-LA) and diamine peroxidase (DAO) concentrations were measured using ELISA kits (Mlbio, Shanghai, China). Also, the commercial ELISA kits were used to assay serum monocyte chemotactic protein 1 (MCP-1), tumor necrosis factor-α (TNF-α), interleukin-1β (IL-1β), and IL-6 levels (Neobioscience, Shenzhen, China). All of the experimental procedures were conducted according to the manuals’ guidelines.

### Immunohistochemical analysis

Immunohistochemical assay was performed as previously described [15, 16], the intestinal tract and the aortic sinus were preserved in 4% paraformaldehyde, embedded in paraffin and dissected to 10 μm sections. Ileal and aortic sinus sections were treated with antigen retrieval (50×citrate sodium buffer, pH 6.0) and endogenous peroxidase inhibition (3% H_2_O_2_ for 15 min), followed by incubation with 1:200 diluted antibodies of ZO-1, occludin, TLR4 and phospho-NF-κB p65 (p-NF-κB p65) (Santa Cruz Biotechnology, Santa Cruz, CA, USA; Abcam, Cambridge, MA, USA) at 4℃ overnight. After washing, the sections were incubated with a peroxidase-conjugated secondary antibody at 37 ℃ for 30 min and labelled with diaminobenzidine, then counter-stained with Harris hematoxylin. The chromogenic slices were finally evaluated under a 200× or 40× magnification optical microscope for image analysis and acquisition.

### Analysis of atherosclerotic lesions

As mentioned before [16], the heart was fixed with optimum cutting temperature compound (Tissue-Tek O.C.T. Compound, SAKURA, Japan) and frozen at -80℃. The heart was placed at -20℃ to make 10 μm thickness frozen sections when necessary. When three valves were present in the aortic sinus, sections were sliced until the valves disappeared. Oil red O staining of frozen sections was used to determine the atherosclerotic lesions in the aortic sinus. After being examined under a microscope with a magnification of 40 times, the size and area of atherosclerotic lesions were finally measured using Image J software (NIH, USA).

### Western blotting

The expression levels of intestinal barrier proteins were quantitatively measured by Western blotting as described previously [15]. The supernatant of a lysed intestinal mucosal solution was taken for protein concentration determination and denaturation, the protein concentration was determined by the BCA Protein Assay Kit (Beyotime, Shanghai, China). The residual supernatant was electrophoresed on a 7.5% SDS-PAGE before being transferred to a nitrocellulose membrane. The transmembrane was then blocked and incubated with polyclonal primary ZO-1 and occludin antibodies (Abcam, Cambridge, MA, USA) with a dilution ratio of 1:1000 at 4 °C overnight. In addition, the protein in the aorta was extracted and subjected to electrophoresis and transmembrane, then the membranes were incubated with polyclonal primary TLR4, p-NF-κB P65 and NF-κB P65 antibodies (Affinity, USA) with a dilution ratio of 1:1000 at 4 °C overnight. Next, the membranes were incubated with 1:10000 diluted secondary antibodies at 25 °C for 2 h. GAPDH antibody was used as the loading control (Santa Cruz Biotechnology, Santa Cruz, CA, USA). Finally, Image J software (NIH, USA) was utilized to quantify and analyze the protein bands.

### Fecal DNA extraction and gut microbiota analysis

As described previously [15], genomic DNA was extracted from feces using the QIAamp DNA Stool Mini Kit (Qiagen, Germany) according to the manufacturer’s recommendations. The V4 region of 16 S rRNA was amplified by PCR using primers 515 F and 806R, and then sequenced by Illumina Hiseq2500 platform. The R Programme Language was used to perform the analysis on the reads of the results. Calculations were done to determine the α-diversity, which included the Observed species, the Shannon index and the Simpson index. For the purpose of determining the level of β-diversity, a Principal Coordinates Analysis (PCoA) was carried out using Bray-Curtis as the distance metric. The test of Kruskal-Wallis and post hoc Dunn were performed to determine the relative abundance of gut microbiota at the phylum and genus levels. For alpha diversity analysis, the ggplot2, vegan, and ggpubr packages were installed firstly. The commands for calculating the Shannon index and Simpson index were in the vegan package, and the commands for calculating the significance of each group were in the ggpubr package; the ggplot2 command was used to draw a picture. To calculate the beta diversity analysis, we used the phyloseq package. For species difference analysis and abundance calculation, the reshape2, ggplot2, ggprism, and plyr packages were installed and used. The correlation coefficient between variables should be calculated by the built-in function cor () in R language and visualized by corrplot package. For data classification, based on the sequencing data obtained by Hiseq/Miseq platform, the Uclust method in QIIME software package was used for OTU cluster analysis according to the sequence similarity level of 97%. Then, based on the Silva reference database, the OTUs of each sample was annotated for species taxonomy. For quality control of sequencing data, we used cutadapt, a sequencing data quality control software, to remove the splice.

Spearman analysis was conducted to clarify the correlation between gut microbiota, lipids, inflammatory factors and intestinal permeability using R software. A corrplot package was used to perform the Spearman correlation.

### Statistical analysis

SPSS statistical software(v19.0)was utilized for all analysis and the values were expressed as the means ± SD. Statistical analysis was conducted through one-way ANOVAs. The differences between groups were compared by SNK and LSD. LSD-t method has the highest sensitivity when comparing the mean of multiple groups, and can find the difference between groups to the maximum extent, which is suitable for preliminary verification trend. At the same time, in order to avoid the risk of Type I errors, a comprehensive pairwise comparison between all groups combined with SNK-q test was performed, so as to clarify the differences between groups and prevent false positives. The R Programming Language was used to analyze the gut microbiota. Pair-to-pair comparison for skewed data were performed using the Wilcox test, and three-group comparison were performed using the Kruskal-Wallis test. Statistical significance was assumed when *p <* 0.05.

## Results

### Basic parameters in ApoE^−/−^ mice

As shown in Table 2, there was no statistically significant difference in body weight gain and daily food intake among the three groups (*p* > 0.05). Similarly, there was no significant difference in the weight of the heart or aorta among the three groups. According to the daily food intake of each mouse and 8 mice per group, the mean of daily calorie intake per group was about 150.4 kcal. In addition, lycopene was incorporated in the high-fat diet at a concentration of 1 g/kg of food (0.1% w/w), the mean of daily lycopene intake in HFD + lycopene group was about 32 mg.

**Table 2:** Basic parameters and biochemical markers measured in ApoE^−/−^ mice

		Group		
		Control	HFD	HFD + LY
Basic parameters	Food intake (g/d) Initial body weight (g) Final body weight (g) Body weight gain (g)	4.00 ± 0.03 22.46 ± 0.99 31.95 ± 1.49 9.49 ± 1.63	4.02 ± 0.12 21.94 ± 0.87 33.59 ± 3.34 11.65 ± 3.39	4.01 ± 0.06 21.95 ± 1.02 30.76 ± 3.97 8.81 ± 3.73
	Heart (standardize to body mass)	0.37 ± 0.32	0.34 ± 0.33	0.38 ± 0.33
	Aorta (standardize to body mass)	0.54 ± 0.38	0.45 ± 0.32	0.61 ± 0.32
	TC (mmol/L)	11.18 ± 3.53^c^	40.06 ± 9.49^c^	25.39 ± 4.27^c^
	TG (mmol/L)	1.11 ± 0.46	2.91 ± 0.83^a^	2.57 ± 1.15
	LDL-C (mmol/L)	21.3 ± 2.82	39.3 ± 3.07^a^	22.61 ± 2.05^b^
	HDL-C (mmol/L)	14.43 ± 1.21^c^	4.84 ± 1.33^c^	10.69 ± 1.01^c^
Serum biochemical marker	TNF-α(pg/ml)	60.23 ± 11.87	85.04 ± 33.34^a^	41.00 ± 9.89^b^
	MCP-1(pg/ml)	93.37 ± 16.87	114.87 ± 20.63^a^	97.87 ± 8.52^b^
	IL-6(pg/ml)	56.38 ± 11.35	93.82 ± 32.34^a^	71.00 ± 15.85^b^
	IL-1β(pg/ml)	65.23 ± 11.84	87.61 ± 24.89^a^	66.02 ± 7.56^b^

Results are mean ± SD, n = 8 per group. One-Way ANOVA statistical method is used for calculation, and LSD and SNK methods are used for inter-group comparison.

Control, low-fat diet; HFD, high-fat diet; HFD + LY, high-fat diet supplemented with lycopene.

^a^ means *p* < 0.05 vs. Control group; ^b^ means *p* < 0.05 vs. HFD group; ^c^ means *p* < 0.05 among three group.

TC, total cholesterol; TG, total triglycerides;

LDL-C, low density lipoprotein cholesterol; HDL-C, high density lipoprotein cholesterol;

TNF-α, tumor necrosis factor-α; MCP-1, monocyte chemotactic protein-1;

IL-6, interleukin-6; IL-1β, interleukin-1β, LY, lycopene.

### Lycopene lowered blood lipids and serum atherogenic inflammatory factors

Serum TC, TG, LDL-C, TNF-α, MCP-1, IL-6 and IL-1β levels were the highest in the HFD group, while the HFD + lycopene group showed significantly reduced levels of serum TC, LDL-C, TNF-α, MCP-1, IL-6 and IL-1β (*p* < 0.05, *p* = 0.02, 0.03, 0.02, 0.01, 0.01, 0.02), but there was no difference in serum TG levels between HFD group and HFD + lycopene group. At the same time, the HFD group had the lowest serum HDL-C concentration, while the HDL-C concentration in HFD + lycopene group was markedly increased (*p* < 0.05, *p* = 0.03) (Table 2).

### Lycopene suppressed Atherosclerosis progression in the aortic sinus

**Fig. 1:**
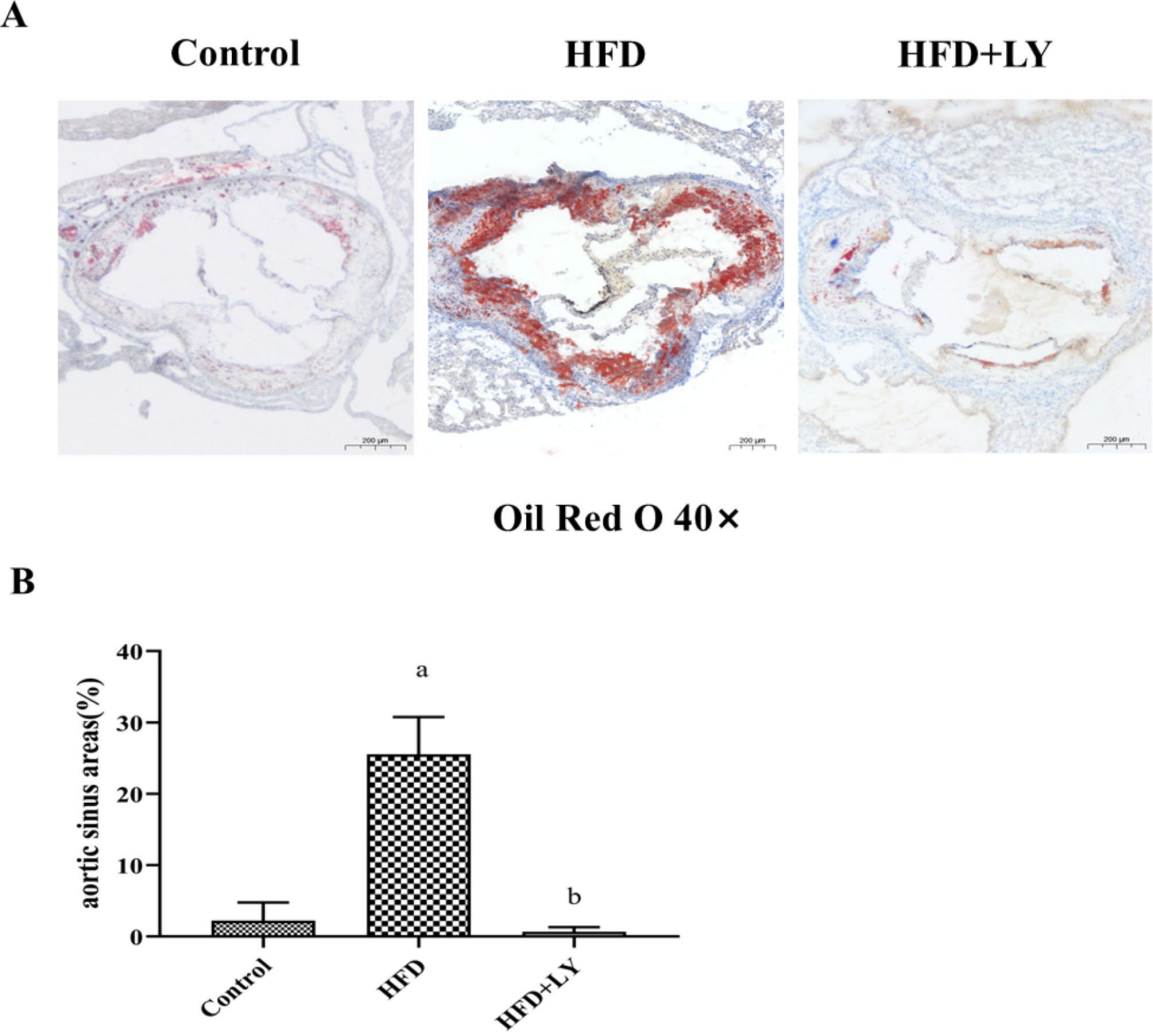
Lycopene supplementation inhibited the atherosclerosis of aortic sinus in HFD-fed ApoE^−/−^ mice. ApoE^−/−^ mice were fed a low-fat diet, a high-fat diet supplemented with or without 0.1% lycopene (w/w) for 19 weeks. (**A**) Oil red O staining of the aortic sinus is presented graphically (40×magnification), as described in Materials and Methods. The larger the area of red plaque, the deeper the atherosclerosis. (**B**) The ratio of the area of atherosclerotic plaque to the area of the aortic sinus was analyzed by Image J software. Data are presented as the mean ± SD (n = 8 per group); ^a^*p* < 0.05 compared with the control group; ^b^*p* < 0.05 compared with the high-fat diet group. HFD, high-fat diet; LY, lycopene

Oil Red O staining was applied to reveal the atherosclerotic lesions in the three groups. As shown in Fig. 1A, the percentage of atherosclerotic lesions was the lowest in the HFD + lycopene group, while the atherosclerotic lesions area in HFD group was larger than that in control group (*p* < 0.05, *p* = 0.01). However, the atherosclerotic lesions area was reduced by 84% in HFD + lycopene group compared to HFD group (Fig. 1B).

### Lycopene modulated the diversity and composition of gut microbiota

**Fig. 2:**
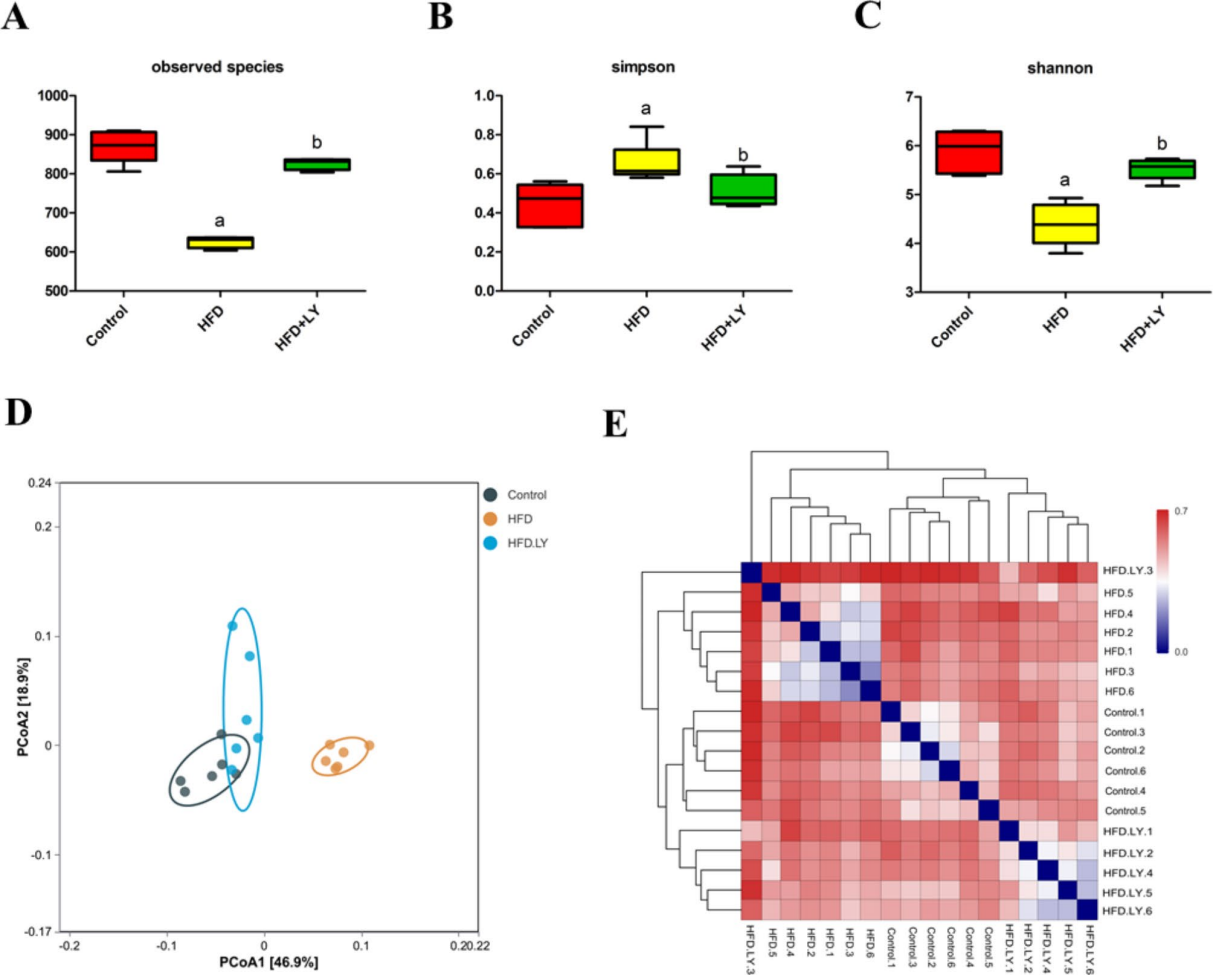
Lycopene supplementation improved the diversity and composition of gut microbiota in HFD-fed ApoE^−/−^ mice. ApoE^−/−^ mice were fed a low-fat diet, a high-fat diet supplemented with or without 0.1% lycopene (w/w) for 19 weeks. (**A, B, C**) The data of the alpha diversity was analyzed by the ggplot2, vegan, and ggpubr packages of R software. Data are presented as the mean ± SD (n = 6 per group); ^a^*p* < 0.05 compared with the control group; ^b^*p* < 0.05 compared with the high-fat diet group. (**D, E**) The data of the beta diversity was analyzed by the phyloseq package of R software. Figure D showed the specificity among three groups. Figure E showed the sample distance of three groups in heatmap; The redder the relationship was closer, while the bluer the relationship was more distant

In the α-diversity analysis, both the Observed species and the Shannon index were reduced in HFD group, while the Simpson index was increased in HFD group. However, lycopene administration improved the diversity of gut microbiota, the Observed species and Shannon index were elevated and the Simpson index was reduced after lycopene treatment (Fig. 2A-C). In the β-diversity analysis, the weighted PCoA diagrams of the three groups were shown in Fig. 2D, which reflected that the composition of gut microbiota in the HFD group was significantly different from the other two groups. Furthermore, a partial overlap was found between HFD + lycopene group and control group, indicating that lycopene supplementation could improve gut microbiota composition induced by HFD. In the heatmap of sample-sample distances, a similar effect was observed (Fig. 2E).

### Lycopene modulated the abundance of gut microbiota

**Fig. 3:**
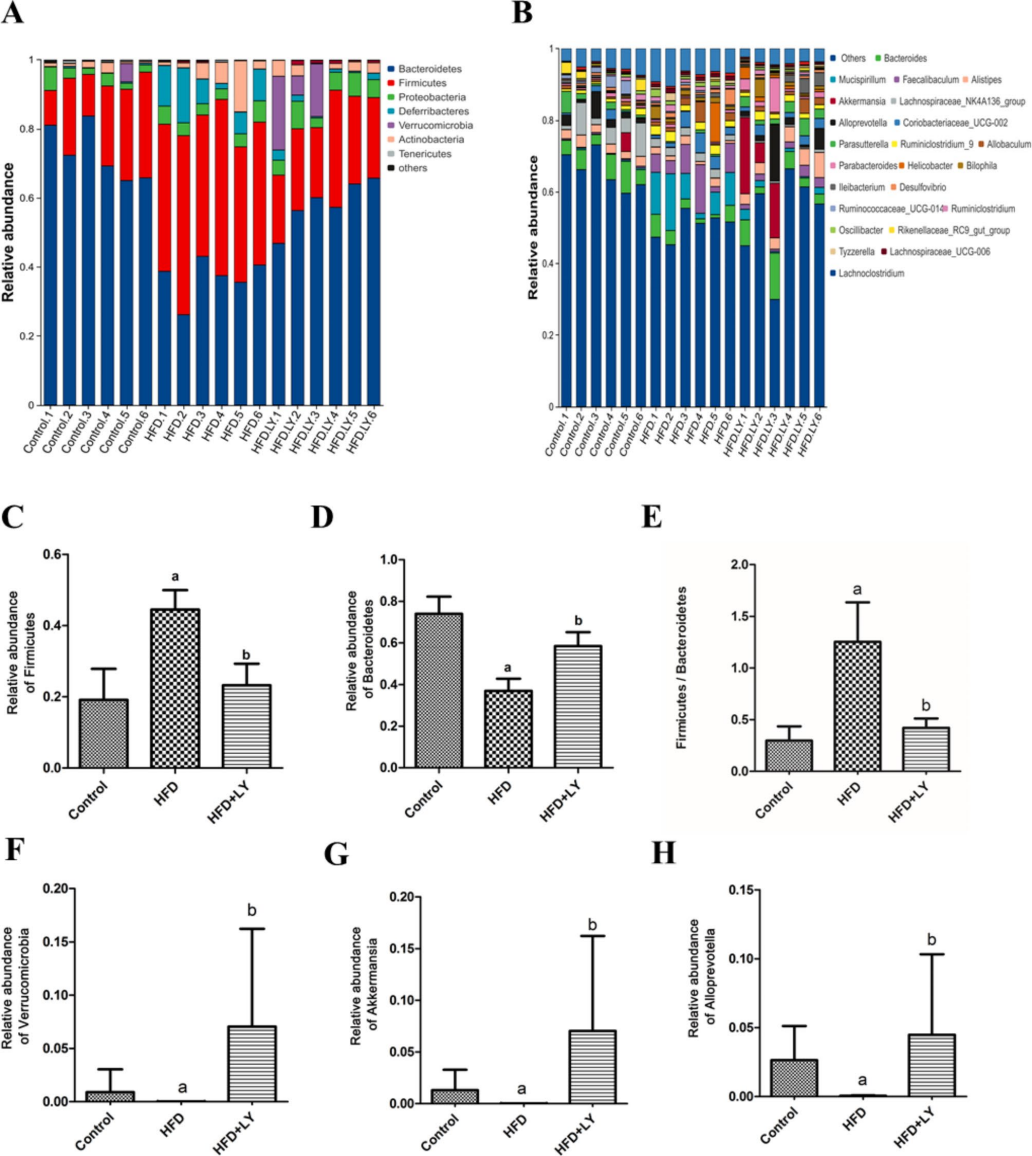
Lycopene supplementation modulated the relative abundance of gut microbiota in HFD-fed ApoE^−/−^ mice. ApoE^−/−^ mice were fed a low-fat diet, a high-fat diet supplemented with or without 0.1% lycopene (w/w) for 19 weeks. (**A, C, D, F**) Different expression of relative abundance of gut microbiota in three groups at phylum level. (**E**) The ratio of Firmicutes/Bacteroides in three groups. Data was analyzed by R software and presented as the mean ± SD (n = 6 per group); ^a^*p* < 0.05 compared with the control group; ^b^*p* < 0.05 compared with the high-fat diet group. (**B, G, H**) Different expressions of relative abundance of gut microbiota in three groups at genus level. Data was analyzed by R software and presented as the mean ± SD (n = 6 per group); ^a^*p* < 0.05 compared with the control group; ^b^*p* < 0.05 compared with the high-fat diet group. HFD, high-fat diet; LY, lycopene. For species difference analysis and abundance calculation, the reshape2, ggplot2, ggprism, and plyr packages of R software were used

To further analyze the alterations in gut microbiota composition, we analyzed the relative abundance of gut microbiota at the phylum and genus levels (Fig. 3A, B). At the phylum level, Firmicutes and Bacteroidetes occupied the largest proportion. Compared to the control group, the relative abundance of Firmicutes was significantly increased and the relative abundance of Bacteroidetes was reduced in HFD group, which was reversed by lycopene (Fig. 3C, D). The Firmicutes/Bacteroides ratio (F/B ratio) was considerably elevated than 1 in HFD group, whereas F/B ratio in the other two groups was significantly less than 1 (*p* < 0.05, *p* = 0.02) (Fig. 3E). Verrucomicrobia was less prevalent in HFD group, whereas its abundance was statistically enhanced in HFD-fed mice supplemented with lycopene (*p* < 0.05, *p* = 0.02) (Fig. 3F). At the genus level, *Akkermansia* and *Alloprevotella* were the lowest in the HFD group while being significantly more prevalent in the HFD + lycopene group (*p* < 0.05, *p* = 0.02) (Fig. 3G, H).

### Lycopene improved intestinal barrier function and reduced intestinal permeability

**Fig. 4:**
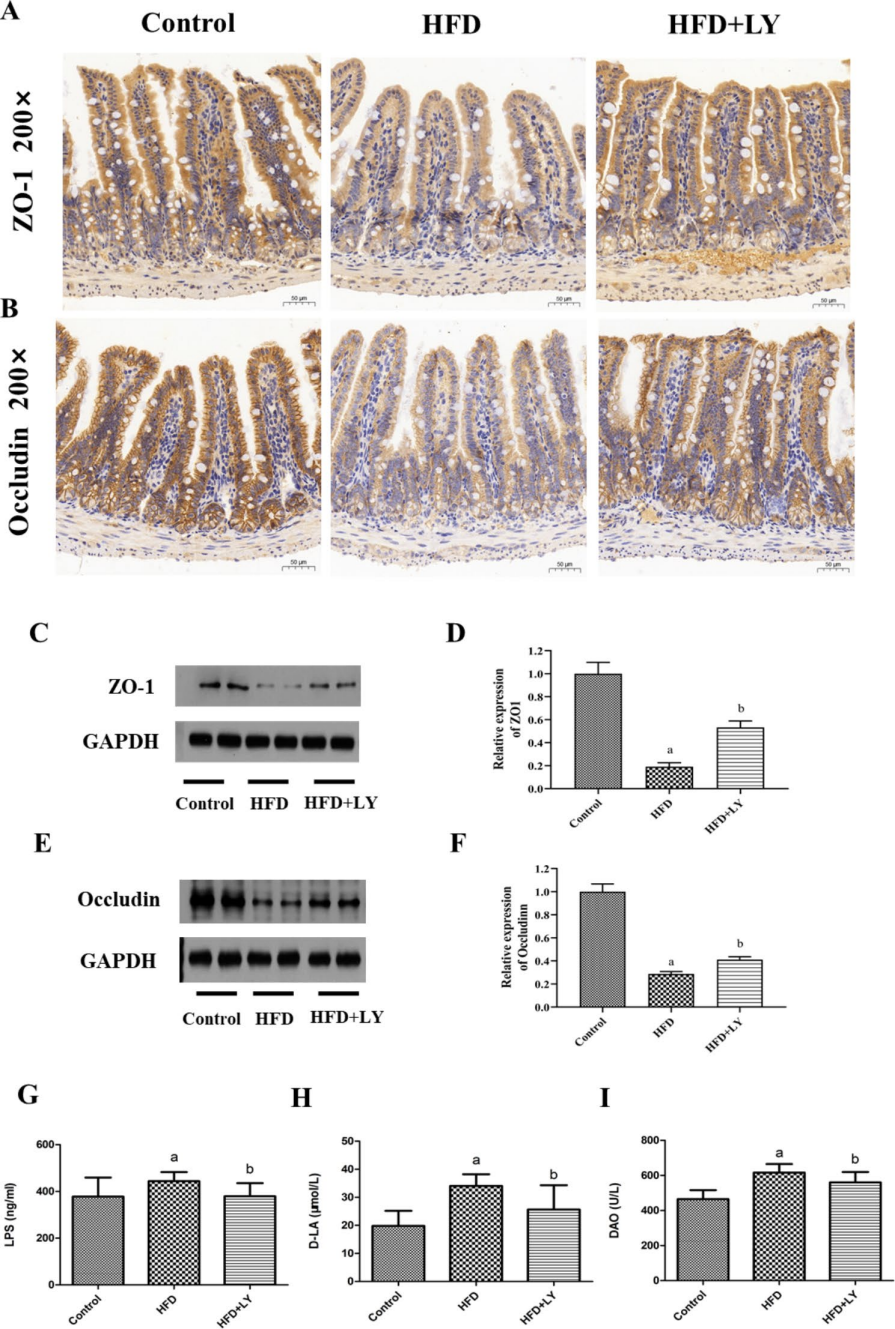
Lycopene supplementation increased intestinal ZO-1 and occludin expression and reduced intestinal permeability in HFD-fed ApoE^−/−^ mice. ApoE^−/−^ mice were fed a low-fat diet, a high-fat diet supplemented with or without 0.1% lycopene (w/w) for 19 weeks. (**A, B**) Immunohistochemical results of ZO-1 and occludin protein expression in the ileum were presented in the form of pictures (200×magnification), as described in Materials and Methods. The browner the color, the higher the protein expression. (**C, D, E, F**) The protein expression of ZO-1 and occludin in the ileum was determined by Western blotting. Results are representative of three independent experiments. It was analyzed by Image J software. Expression values were normalized to housekeeping gene GAPDH, and expression in the control group was set to 1. Data are presented as the mean ± SD (n = 8 per group); ^a^*p* < 0.05 compared with the control group; ^b^*p* < 0.05 compared with the high-fat diet group. (**G, H, I**) Intestinal permeability index such as LPS, D-LA and DAO was analyzed by SPSS software. One-Way ANOVA statistical method was used for calculation, and LSD and SNK methods were used for inter-group comparison. Data are presented as the mean ± SD (n = 8 per group); ^a^*p* < 0.05 compared with the control group; ^b^*p* < 0.05 compared with the high-fat diet group. HFD, high-fat diet; LY, lycopene

The intestinal barrier function can be reflected by the expression levels of epithelial tight junction protein ZO-1 and occludin. Immunohistochemical analysis and Western blotting revealed that the expression levels of intestinal ZO-1 and occludin in the HFD group were statistically decreased compared with the control group. In contrast, HFD + lycopene group showed higher levels of intestinal ZO-1 and occludin expression (Fig. 4A-F), reflecting an improved intestinal barrier function. Moreover, the concentrations of serum LPS, D-LA and DAO, which are the biomarkers of intestinal permeability, were noticeably increased in the HFD group, while the HFD + lycopene group showed decreased levels (*p* < 0.05, *p* = 0.02, 0.01, 0.03) (Fig. 4G-I), reflecting a reduced intestinal permeability.

### Lycopene inhibited inflammation in the aortic sinus

**Fig. 5:**
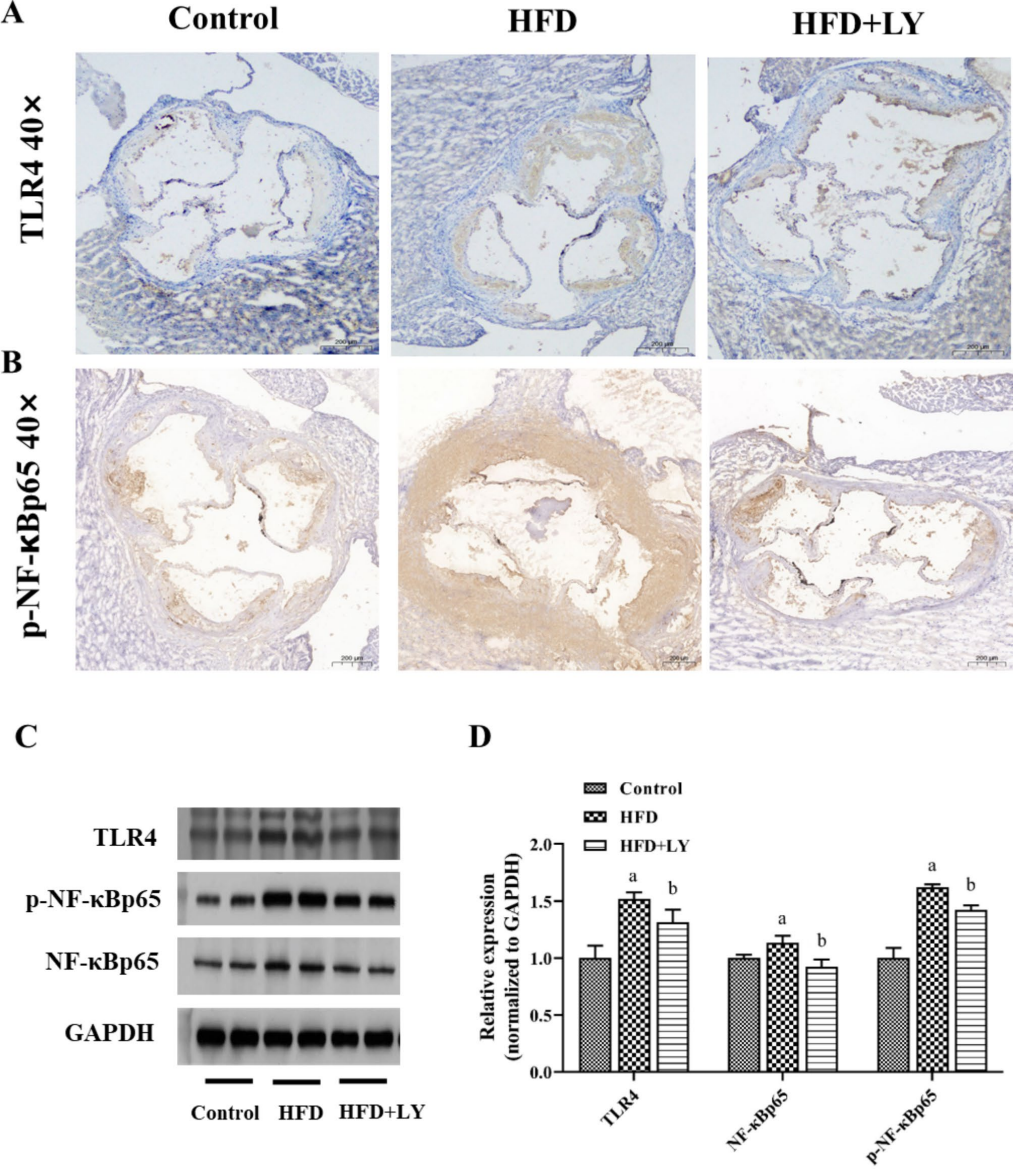
Lycopene supplementation attenuated the inflammation of aortic sinus in HFD-fed ApoE^−/−^ mice. ApoE^−/−^ mice were fed a low-fat diet, a high-fat diet supplemented with or without 0.1% lycopene (w/w) for 19 weeks. (**A, B**) Immunohistochemical results of TLR4 and p-NF-κB P65 protein expression in the aortic sinus were presented graphically (40×magnification), as described in Materials and Methods. The browner the color, the higher the protein expression. (**C, D**) The protein expression levels of aortic TLR4, p-NF-ĸBp65 and NF-ĸBp65 were determined by Western blotting. Results are representative of three independent experiments. It was analyzed by Image J software. Expression values were normalized to housekeeping gene GAPDH, and expression in the control group was set to 1. Data are presented as the mean ± SD (n = 8 per group); ^a^*p* < 0.05 compared with the control group; ^b^*p* < 0.05 compared with the high-fat diet group. HFD, high-fat diet; LY, lycopene

Elevated LPS enters the circulation and binds to TLR4, which in turn stimulates the TLR4/NF-κB signaling pathway, thereby activating various inflammatory factors and potentially resulting in atherosclerosis. As illustrated in Fig. 5A, B, the

immunohistochemical analysis showed that the expression of inflammatory protein TLR4 and p-NF-κB p65 in the aortic sinus was markedly increased in the HFD group, which was significantly reduced in HFD + lycopene group compared to the HFD group. There was little difference between the control group and HFD + lycopene group. In Western blotting analysis,

the expression levels of aortic TLR4, p-NF-κB p65 and NF-κB p65 were considerably higher in HFD group. Surprisingly, the expression levels of the three proteins in the HFD + lycopene group were decreased, while the expression levels of the control group were the lowest (Fig. 5C, D). Accordingly, the serum levels of inflammatory cytokines, such as TNF-α, MCP-1, IL-6 and IL-1β, were significantly reduced in HFD + lycopene group compared with HFD group (*p* < 0.05, *p* = 0.01, 0.02, 0.02, 0.03) (Table 2).

### Spearman’s correlations analysis between the gut microbiota and the biochemical markers

**Fig. 6:**
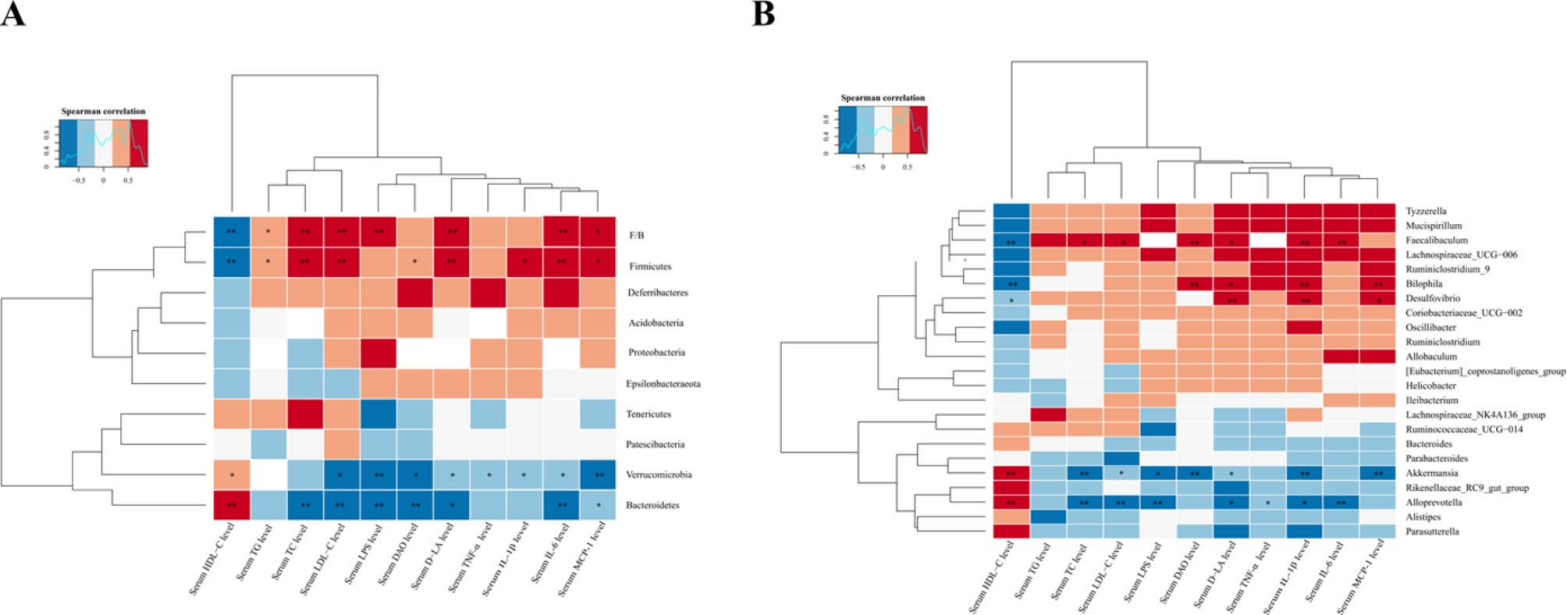
Spearman’s correlations between the gut microbiota and the biochemical markers. (**A**) The correlations between the gut microbiota and the biochemical markers at the phylum level. (**B**) The correlations between the gut microbiota and the biochemical markers at the genus level. The colour intensity represents the degree of the associations between the gut microbiota and the biochemical parameters, red represents positive correlation and blue indicates negative correlations. n = 6 per group, significant correlations are marked by ^*^p < 0.05 and ^**^p < 0.01. HFD, high-fat diet; LY, lycopene

Spearman’s correlations analysis also showed that different gut microbiota was associated with serum lipid, inflammatory factors and intestinal permeability indexes. At the phylum level, Firmicutes were positively correlated with serum D-LA, DAO, MCP-1, IL-1β, IL-6, TC, TG and LDL-C levels, while Firmicutes were negatively correlated with serum HDL-C levels; Bacteroides were negatively correlated with serum LPS, DAO, D-LA, MCP-1, IL-6, TC and LDL-C levels, but Bacteroides were positively correlated with serum HDL-C levels. Similarly, Verrucomicrobia showed the same trend, but no negative correlation with TC was observed. The Firmicutes/Bacteroides ratio was positively correlated with serum LPS, D-LA, MCP-1, IL-6, TC, TG and LDL-C levels, while serum HDL-C levels were negatively correlated with the Firmicutes/Bacteroides ratio (Fig. 6A).

At the genus level, *Akkermansia* and *Alloprevotella* were negatively correlated with serum D-LA, DAO, MCP-1, IL-6, IL-1β, TNF-α, TC, TG and LDL-C levels, while the serum HDL-C levels showed a positive correlation (Fig. 6B).

## Discussion

In this study, lycopene treatment significantly improved the gut microbiota dysbiosis, reduced the ratio of Firmicutes/Bacteroides and elevated the relative abundance of gut microbiota such as Verrucomicrobia, *Akkermansia* and *Alloprevotella*, which were associated with reduced inflammation and enhanced intestinal barrier function. Moreover, lycopene administration improved the intestinal barrier function and alleviated intestinal permeability, then reduced serum LPS levels and inhibited cardiovascular inflammation through inactivating the TLR4/NF-κB pathway, thereby inhibiting the progression of atherosclerosis. Our finding provides a novel insight into the role of lycopene in preventing atherosclerosis and the underlying mechanism associated with the gut-heart axis.

As a phytochemical, lycopene has been shown to regulate lipid metabolism and reduce lipid levels in vivo [17–19]. Higher lycopene intake has demonstrated a stronger protective effect on the heart [18]. As Chiva-Blanch et al. reported, an inverse correlation between the circulating levels of lycopene and the progression of atherosclerosis was observed in newly diagnosed patients with type 2 diabetes [20]. These results suggest that as a part of healthy diet, lycopene supplementation can inhibit atherosclerosis formation. In addition, lycopene supplementation has been shown to prevent the development of atherosclerosis in the carotid arteries of middle-aged and old people [13]. A recent study indicates that daily supplementation of lycopene for three months can inhibit the formation of atherosclerotic plaque in male rats [21]. Our previous study also showed that lycopene administration could lower serum lipid levels and suppress the formation of atherosclerotic plaque in the aortic sinus of HFD-fed ApoE^−/−^ mice through inhibiting intestinal cholesterol absorption [19], which was consistent with our current study. Considering the poor absorption in the gut and low concentration of lycopene in the blood [22], its effect in the intestinal tract might be of more physiological implications than its systemic effects.

A number of evidence has reported that the gut microbiota has a crucial implication in the pathogenesis of atherosclerosis [4, 5]. Alterations in the gut microbiota caused either by the diet or other factors promote the progression of atherosclerosis via the gut-heart axis [23]. The high-fat diet has been shown to alter the composition and function of the gut microbiota in mice [6, 24]. A high-fat diet caused an increase in the ratio of Firmicutes/Bacteroides and a decrease in the relative abundance of Verrucomicrobia in the gut [24, 25], which are related to the gut microbiota disorder and the impairment of intestinal barrier function. Previous study showed that a mixture of lycopene and dark chocolate could regulate the gut microbiota in moderately obese people [26]. A recent research also reported that lycopene supplementation improved the gut microbiota dysbiosis and intestinal barrier function in weaned piglets [12]. Excitingly, our current study demonstrated that lycopene significantly improved the species diversity of gut microbiota, decreased the ratio of Firmicutes/Bacteroides and increased the relative abundance of Verrucomicrobia in HFD-fed ApoE^−/−^ mice. It has been reported that the improvement in the ratio of Firmicutes/Bacteroides is associated with the restoration of intestinal barrier function and reduced serum LPS levels [25]. Similarly, increased Verrucomicrobia is thought to repair the intestinal barrier function and alleviate endotoxemia and systemic inflammation [27]. More importantly, at the genus level, lycopene administration was found to significantly increase the relative abundance of *Akkermansia* and *Alloprevotella* in the gut. Previous studies demonstrated that *Akkermansia* and *Alloprevotella* could restore the intestinal barrier function, eliciting a beneficial immune response [28, 29]. There is considerable evidence proving that intestinal *Akkermansia* prevalence is negatively correlated with inflammation-related disorders, including diabetes, obesity and atherosclerosis [28, 30, 31]. Intestinal *Alloprevotella* has also been shown to play a similar role in preventing these inflammatory diseases [15, 32, 33]. Furthermore, supplementation with *Akkermansia* could stimulate the expression of intestinal tight junction proteins such as ZO-1 and occludin and reduce intestinal permeability and serum endotoxin levels, thereby alleviating endotoxin-induced inflammation and preventing atherosclerosis in ApoE^−/−^ mice fed western diet [28]. Likewise, our results also found that higher abundance of *Akkermansia* and *Alloprevotella* was associated with lower levels of serum LPS and inflammatory markers, such as IL-6, IL-1β, TNF-α and MCP-1. Additionally, *Akkermansia* and *Alloprevotella* were negatively correlated with serum lipid levels and intestinal permeability indicators. These results suggest that lycopene treatment restores the gut *Akkermansia* and *Alloprevotella* communities and improve the intestinal barrier function in HFD-fed ApoE^−/−^ mice, leading to reduced intestinal permeability, endotoxin levels and inflammatory response.

The intestinal epithelial barrier serves as the primary defence line of body against the external environment. The gut microbiota dysbiosis may disrupt the tight junctions of intestinal epithelium cells, thus attenuating the intestinal barrier function. The main tight junction proteins such as ZO-1 and occludin play important roles in the regulation of intestinal permeability and in the maintenance of intestinal barrier integrity [34]. Reduced expression of intestinal tight junction proteins such as ZO-1 and occludin will disrupt the intestinal barrier by causing epithelial cell death, leading to increased intestinal permeability. A high-fat diet has been reported to cause gut microbiota dysbiosis and reduced the expression levels of intestinal tight junction proteins, resulting in increased intestinal permeability and serum endotoxin levels ^6^. Our current study also showed that the high-fat diet caused gut microbiota dysbiosis and significantly reduced the protein expression of ZO-1 and occludin, severely disrupting the tight junctions among intestinal epithelium cells. Meanwhile, serum LPS, D-lactate and DAO levels, which are the biomarkers of intestinal permeability [35], were also increased with the decreased expression of intestinal tight junction protein. However, lycopene supplementation could improve gut microbiota dysbiosis and increase ZO-1 and occludin protein expression, then effectively reduce intestinal permeability and serum LPS levels, as well as LPS entry into the heart.

LPS is also a natural ligand of TLR4 and can activate the TLR4-related inflammatory signaling pathway, resulting in the release of inflammatory cytokines [36]. TLR4 belongs to the TLR family and is considered as a linking molecule between the gut microbiota, endotoxemia and atherosclerosis [37]. TLR4 is highly expressed in atherosclerotic plaque and is associated with elevated serum LPS levels and impaired intestinal barrier [4]. Genetic deletion of TLR4 in ApoE^−/−^ mice was related to the reduced serum LPS levels and macrophage inflammatory response and atherosclerotic lesions in the aorta [38]. The human studies also demonstrated that the elevated expression of TLR4 was associated with inflammatory activation in atherosclerosis, and promoted the development of atherosclerosis [39]. The gut microbiota dysbiosis can enhance the permeability of intestinal epithelial barrier and cause endotoxin LPS accumulation in the heart. The binding of LPS to TLR4 can activate its downstream pathway NF-κB. The activation of NF-κB leads to the release of several pro-inflammatory cytokines including IL-6, IL-1β, MCP-1 and TNF-α [5]. Pro-inflammatory cytokines, such as IL-6, IL-1β, MCP-1 and TNF-α, are involved in the pathogenesis of atherosclerosis through inducing inflammatory cascades in the heart [24, 40, 41]. A recent study has shown that tomato powder containing 2.39 mg lycopene can reduce the mRNA expression levels of IL-6, IL-1β, TNF-α and MCP-1 in high-fat diet-fed mice [42]. Our previous studies in RAW264.7 macrophages also found that lycopene treatment inhibited LPS-induced inflammatory response through suppressing the trafficking of TLR4 to lipid rafts and the subsequent activation of NF-κB pathway [10, 11], leading to reduced inflammatory cytokines production. Consistently, our current study also showed that lycopene supplementation improved gut microbiota dysbiosis and reduced serum endotoxin LPS levels, subsequently inhibited the activation of TLR4/NF-κB signaling pathway in the heart and decreased inflammatory cytokines release, ultimately achieving an anti-atherosclerotic effect in HFD-fed ApoE^−/−^ mice.

## Conclusion

In summary, our results indicated that the protective effect of lycopene against atherosclerosis induced by high-fat diet and further revealed that its mechanism might be its prebiotic effect on maintaining gut microbiota homeostasis and improving intestinal barrier function, consequently reducing serum lipopolysaccharide-triggered inflammatory response in the heart. Overall, this study provides a novel insight into the anti-atherosclerotic potential and mechanism of phytochemicals lycopene and the correlation between the gut microbiota and the heart, which lays a foundation for the clinic application of lycopene to protect against atherosclerosis and inflammation-related diseases.

## Data Availability

The datasets used during the present study are available from the corresponding author upon reasonable request.

## Abbreviations

ApoE^−/−^: Apolipoprotein E knockout
ALT: Alanine transferase
AST: Aspartic transaminase
D-LA: D-lactic acid
DAO: Diamine peroxidase
F/B ratio: Firmicutes / Bacteroides ratio
HDL-C: High density lipoprotein cholesterol
HFD: High-fat diet
IL-1β: interleukin-1β
LDL-C: Low density lipoprotein cholesterol
LPS: Lipopolysaccharide
MCP-1: Monocyte chemotactic protein-1
NF-κB: Nuclear factor-kappa B
PCoA: Principal coordinates analysis
p-NF-κB p65: Phospho-NF-κB p65
TC: Total cholesterol
TG: Triglyceride
TLR4: Toll-like receptor 4
TNF-α: Tumor necrosis factor-α
ZO-1: Zonula occludens-1
